# Comparative genomic analysis uncovers 3 novel loci encoding type six secretion systems differentially distributed in *Salmonella *serotypes

**DOI:** 10.1186/1471-2164-10-354

**Published:** 2009-08-04

**Authors:** Carlos J Blondel, Juan C Jiménez, Inés Contreras, Carlos A Santiviago

**Affiliations:** 1 Departamento de Bioquímica y Biología Molecular, Facultad de Ciencias Químicas y Farmacéuticas, Universidad de Chile, P.O. Box 174, Correo 22, Santiago, Chile; 2 Programa de Microbiología y Micología, Instituto de Ciencias Biomédicas, Facultad de Medicina, Universidad de Chile, Av. Independencia 1027, Santiago, Chile

## Abstract

**Background:**

The recently described Type VI Secretion System (T6SS) represents a new paradigm of protein secretion in bacteria. A number of bioinformatic studies have been conducted to identify T6SS gene clusters in the available bacterial genome sequences. According to these studies, *Salmonella *harbors a unique T6SS encoded in the *Salmonella *Pathogenicity Island 6 (SPI-6). Since these studies only considered few *Salmonella *genomes, the present work aimed to identify novel T6SS loci by *in silico *analysis of every genome sequence of *Salmonella *available.

**Results:**

The analysis of sequencing data from 44 completed or in progress *Salmonella *genome projects allowed the identification of 3 novel T6SS loci. These clusters are located in differentially-distributed genomic islands we designated SPI-19, SPI-20 and SPI-21, respectively. SPI-19 was identified in a subset of *S. enterica *serotypes including Dublin, Weltevreden, Agona, Gallinarum and Enteritidis. In the later, an internal deletion eliminated most of the island. On the other hand, SPI-20 and SPI-21 were restricted to *S. enterica *subspecies *arizonae *(IIIa) serotype 62:z4,z23:-. Remarkably, SPI-21 encodes a VgrG protein containing a C-terminal extension similar to S-type pyocins of *Pseudomonas aeruginosa*. This is not only the first evolved VgrG described in *Salmonella*, but also the first evolved VgrG including a pyocin domain described so far in the literature. In addition, the data indicate that SPI-6 T6SS is widely distributed in *S. enterica *and absent in serotypes Enteritidis, Gallinarum, Agona, Javiana, Paratyphi B, Virchow, IIIa 62:z4,z23:- and IIIb 61:1,v:1,5,(7). Interestingly, while some serotypes harbor multiple T6SS (Dublin, Weltvreden and IIIa 62:z4,z23:-) others do not encode for any (Enteritidis, Paratyphi B, Javiana, Virchow and IIIb 61:1,v:1,5,(7)). Comparative and phylogenetic analyses indicate that the 4 T6SS loci in *Salmonella *have a distinct evolutionary history. Finally, we identified an orphan Hcp-like protein containing the Hcp/COG3157 domain linked to a C-terminal extension. We propose to designate this and related proteins as "evolved Hcps".

**Conclusion:**

Altogether, our data suggest that (i) the *Salmonella *T6SS loci were acquired by independent lateral transfer events and (ii) evolved to contribute in the adaptation of the serotypes to different lifestyles and environments, including animal hosts. Notably, the presence of an evolved VgrG protein related to pyocins suggests a novel role for T6SS in bacterial killing. Future studies on the roles of the identified T6SS loci will expand our knowledge on *Salmonella *pathogenesis and host specificity.

## Background

The recently described Type VI Secretion System (T6SS) represents a new paradigm of protein secretion in bacteria maintaining pathogenic or symbiotic interactions with eukaryotic organisms (reviewed in references [1-4]). In this context, T6SSs have been linked to a variety of functions such as adherence, cytotoxicity, host-cell invasion, biofilm formation, survival within macrophages, and persistence within the host [2,4].

T6SS are encoded in loci initially known as IAHP (IcmF associated homologous protein) clusters. These gene clusters were characterized by the presence of approximately 15 ORFs surrounding a homolog of IcmF, a protein associated with type IV secretion in *Legionella pneumophila *[5-7]. In addition to the variable genetic architecture presented by IHAPs in several microbial genomes, low level of sequence identity between components of the system in different bacteria has hampered the identification of new T6SS loci. In spite of this, *in silico *analyses identified a set of 13 conserved proteins defined as the T6SS "core components" [8]. These components carry a distinct COG ID (Cluster of Orthologous Groups of proteins), most of which are unique for T6SS function [1,8]. In addition, there are 15 conserved accessory proteins widely distributed among T6SS loci that include specific transcriptional and post-transcriptional regulators [8].

Most T6SS core components correspond to structural elements of the secretion machinery. The DotU and IcmF homologs (COG3523 and COG3455, respectively) are conserved inner membrane proteins essential for secretion [9,10]. A role for IcmF and DotU homologs in membrane stabilization of the T6SS apparatus has been proposed based on observations made for homolog proteins involved in stabilization of the T4SS apparatus in *L. pneumophila *[6]. The ClpV homologs (COG0542) belong to the AAA+ family of ATPases and are hypothesized to energize the system, enabling protein secretion [9,11,12]. Recently, it has been reported that *V. cholerae *ClpV interacts through its N-terminal domain with a tubular structure formed by VipA (IglA, COG3516) and VipB (IglB, COG 3517), two conserved and essential T6SS components. Remodeling of VipA/VipB tubules by ClpV-mediated threading is crucial for type VI protein secretion [13]. In addition, it has been recently proposed that the VipA/VipB structure corresponds to a structural and possibly functional homolog of the tail sheath in bacteriophage T4, as both structures present similar dimensions, symmetry and overall organization [14].

Several other T6SS core components resemble proteins of bacteriophage origin. This is the case of the hemolysin coregulated protein Hcp (COG3157), the valine-glycine repeat protein VgrG (COG3501) and the gp25-like protein (COG3518), which are homologs to components of the tail and baseplate in bacteriophage T4 [14,15]. Interestingly, both Hcp and VgrG appear to be structural and secreted components. Thus, the detection of both proteins in culture supernatants has become an indicator of T6SS functionality [10,16,17]. Some VgrG proteins known as "evolved VgrGs" present a C-terminal extension including "effector domains" that have been linked to a variety of functions, such as crosslinking of host actin, degradation of the peptidoglycan layer, and ADP-ribosylation of host proteins [4,16]. The evolved VgrGs have been identified in a limited number of bacteria and are usually encoded outside T6SS loci, scattered throughout the genome.

Following the initial characterization of IAHP clusters [5], several *in silico *analyses have identified T6SS loci in bacterial genome sequences available. In 2008, a screen for homologs to the conserved T6SS components VipA/IglA and VipB/IglB identified 37 T6SS loci in 29 bacteria [1]. Later that year, a similarity search for orthologs of T6SS components described in *Vibrio cholerae*, *Pseudomonas aeruginosa *and *Burkholderia mallei *identified T6SS loci in 42 bacterial species [18]. Even though both analyses greatly expanded the number of T6SS loci reported, they were based on the detection of homologs to T6SS components present in only three organisms, overlooking the existence of phylogenetically distant T6SS loci. A more recent study addressed this limitation by analyzing the presence of conserved protein domains rather than orthologous proteins, to identify 176 T6SS loci from 92 different bacteria [8].

The first report for a T6SS in *Salmonella *corresponds to the genetic characterization of the *Salmonella *Pathogenicity Island 6 (SPI-6), formerly known as SCI (*Salmonella enterica *centisome 7 island), which is adjacent to the tRNA-encoding gene *aspV *[19]. SPI-6 is a ~47 kb genomic island presenting a mosaic structure characterized by the presence of the *saf *and *tcf *fimbrial operons. Analysis of the region upstream of the fimbrial operons revealed numerous ORFs encoding putative periplasmic, outer membrane and secreted proteins, suggesting that SPI-6 encoded a novel secretion system [19]. After the classification of SPI-6 as an IAHP locus [5] and the discovery of T6SSs in *V. cholerae *and *P. aeruginosa *[9,20] it was clear that SPI-6 encoded a T6SS, although there is no experimental evidence on the functionality of this system. Conflicting reports associate SPI-6 T6SS with *Salmonella *pathogenesis. A *S*. Typhimurium mutant in STM0285 (IcmF homolog) presented an increased intracellular growth in macrophages and was hypervirulent in BALB/c mice [21]. On the other hand, mutations in STM0272 (ClpV homolog) and STM0291 (Rhs element) have been reported to cause a ~30% reduction in the ability of *S*. Typhimurium to replicate in macrophages [22]. In addition, overexpression of a dominant negative version of STM0272 impaired the ability of *S*. Typhimurium to invade epithelial cells [12].

Despite the recent advances in our understanding of the structural and genomic organization of T6SS gene clusters, every *in silico *analysis performed so far to identify these loci in bacteria has taken into account a limited number of the currently available *Salmonella *genome sequences. Considering that many bacterial species harbor multiple T6SS in their genomes, it is plausible to think that there could be more than one T6SS in the genus *Salmonella *in addition to SPI-6 T6SS waiting to be uncovered. In the present work we performed a genome-wide *in silico *analysis of all currently available *Salmonella *genome sequences to identify T6SS loci. Our analysis revealed the presence of 3 novel T6SS gene clusters encoded in differentially-distributed genomic islands presenting distinctive evolutionary histories.

## Results and Discussion

### Identification of T6SS gene clusters in *Salmonella*

Representatives of the 13 T6SS core components recently defined by Boyer and coworkers [8] were used as baits to identify T6SS loci by sequential BLASTN, BLASTP and TBLASTX searches using all publicly available sequences from 44 *Salmonella *genome sequencing projects (completed or in progress). The data analyzed included both chromosome and plasmid sequences covering 24 different serotypes of *Salmonella enterica *(22 in subspecies I, 1 in subspecies IIIa and 1 in subspecies IIIb) (Additional file 1).

**Figure 1:**
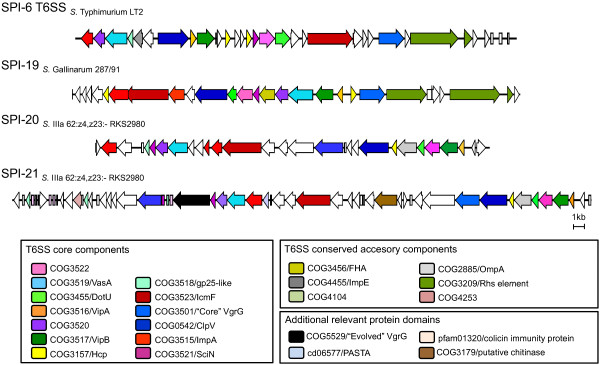
**Gene organization of T6SS gene clusters in *Salmonella***. Schematic representation of the T6SS loci identified in *Salmonella*, including the previously described T6SS encoded in SPI-6. One representative of each T6SS-encoding island is shown. ORFs are represented as blocked arrows showing the direction of their transcription. Conserved core, accessory and additional components are represented with a different color.

To maximize the power of the screen, representatives of each core component from 3 different organisms belonging to each of the different branches in phylogenetic trees previously defined for T6SS loci [1,8] were used as baits. The analysis revealed the presence of 3 novel T6SS loci in addition to the gene cluster encoded within SPI-6 (Table 1). The novel T6SS gene clusters are located in 3 genomic islands we have designated SPI-19, SPI-20 and SPI-21, respectively. These islands are differentially distributed among the *Salmonella *serotypes analyzed and contain each of the 13 T6SS core components described to date (Figure 1). Most *Salmonella *present a unique T6SS encoded either by SPI-6 or SPI-19. On the other hand, a limited number of *Salmonella *carry two T6SS, encoded either by SPI-6 and SPI-19 or by SPI-20 and SPI-21. Finally, no T6SS locus was identified in the available genomes of *S*. *enterica *subspecies *enterica *(I) serotypes Paratyphi B, Virchow and Javiana, and *S*. *enterica *subspecies *diarizonae *(IIIb) serotype 61:1,v:1,5,(7).

**Table 1:** Distribution of T6SS loci in *Salmonella*

				T6SS locus			
				
Genus	Species	Subspecies	Serotype	SPI-6	SPI-19	SPI-20	SPI-21
*Salmonella*	*enterica*	*enterica *(I)	Typhi	X			
*Salmonella*	*enterica*	*enterica *(I)	Typhimurium	X			
*Salmonella*	*enterica*	*enterica *(I)	Paratyphi A	X			
*Salmonella*	*enterica*	*enterica *(I)	Paratyphi C	X			
*Salmonella*	*enterica*	*enterica *(I)	Kentucky	X			
*Salmonella*	*enterica*	*enterica *(I)	Newport	X			
*Salmonella*	*enterica*	*enterica *(I)	Heidelberg	X			
*Salmonella*	*enterica*	*enterica *(I)	Saintpaul	X^a^			
*Salmonella*	*enterica*	*enterica *(I)	Schwarzengrund	X			
*Salmonella*	*enterica*	*enterica *(I)	Tennessee	X			
*Salmonella*	*enterica*	*enterica *(I)	4, [5] ,12:i:-	X			
*Salmonella*	*enterica*	*enterica *(I)	Hadar	X			
*Salmonella*	*enterica*	*enterica *(I)	Infantis	X			
*Salmonella*	*enterica*	*enterica *(I)	Choleraesuis	X			
*Salmonella*	*enterica*	*enterica *(I)	Weltevreden	X	X		
*Salmonella*	*enterica*	*enterica *(I)	Dublin	X	X		
*Salmonella*	*enterica*	*enterica *(I)	Gallinarum		X		
*Salmonella*	*enterica*	*enterica *(I)	Agona		X		
*Salmonella*	*enterica*	*enterica *(I)	Enteritidis		X^b^		
*Salmonella*	*enterica*	*enterica *(I)	Paratyphi B				
*Salmonella*	*enterica*	*enterica *(I)	Virchow				
*Salmonella*	*enterica*	*enterica *(I)	Javiana				

*Salmonella*	*enterica*	*arizonae *(IIIa)	62:z4,z23:-			X	X

*Salmonella*	*enterica*	*diarizonae *(IIIb)	61:1,v:1,5,(7)				

^a ^SPI-6 in strain SARA29 presents an internal deletion of ~15 kb. In contrast, strain SARA23 harbors a full version of the island.

^b ^SPI-19 in serotype Enteritidis presents an internal deletion of ~24 kb.

### SPI-19: a second T6SS gene cluster in *S. enterica *subspecies *enterica* (I)

SPI-19 is a novel T6SS locus present in a subset of serotypes belonging to *S*. *enterica *subspecies *enterica *(I), including Dublin, Weltevreden, Agona, Gallinarum and Enteritidis. Remarkably, serotypes Dublin and Weltevreden also carry the T6SS encoded in SPI-6, while serotype Enteritidis only presents a truncated version of SPI-19 (see below). The ORFs, product sizes, conserved protein domains and genome coordinates of SPI-19 genes in serotypes Dublin, Weltevreden, Agona, Gallinarum and Enteritidis are detailed in Additional file 2.

The island corresponds to a ~45 kb element located in the vicinity of a gene cluster encoding putative proteins involved in sugar transport and utilization (STM1127 to STM1133 in serotype Typhimurium), and is not directly linked to a tRNA-encoding gene (see below). Although the average G+C content of the island is similar to that of the whole genome (e.g., 54.3% versus 52.2% in serotype Gallinarum), the nucleotide content is not evenly distributed. For instance, regions of SPI-19 in serotype Gallinarum encoding Hcp-like proteins and proteins of unknown function presents a low G+C content (34.5%), while the rest of the island presents a G+C content significantly higher (59.1%).

SPI-19 encodes ~30 ORFs, including each T6SS core component (Figure 1 and Additional file 2). Notably, the island includes two ORFs encoding Hcp-like proteins that only share 27% of sequence identity (e.g., SG1029 and SG1043 in serotype Gallinarum). We refer to these proteins as Hcp-1 and Hcp-2, respectively (Figure 1 and Additional file 2). Hcp-1 is encoded in a putative operon that includes most of the T6SS functions. On the other hand, Hcp-2 is encoded upstream of a VgrG homolog at the opposite end of the island. This VgrG protein does not present a C-terminal extension or a putative effector domain. Additionally, the island includes 2 distantly related ImpA homologs (e.g., SG1030 and SG1032 in serotype Gallinarum), as reported for other T6SS loci [18]. SPI-19 does not encode transcriptional and post-transcriptional regulators present in others T6SS gene clusters [11,20], but encodes a protein containing the forkhead-associated (FHA) domain. It is known that an FHA-domain containing protein interacts with a Ser/Thr kinase (PpkA) and a phosphatase (PppA) to regulate type VI secretion in *P. aeruginosa *[11]. Although SPI-19 does not encode kinases and phosphatases, the possible interaction of the FHA-domain containing protein with other kinases and phosphatases to regulate secretion by this T6SS cannot be ruled out. In addition to T6SS-related components, a small "Sel1-like repeat protein" was identified in SPI-19 (Additional file 2). Serotypes Agona and Weltevreden encode a full version of the protein (SeAg_B1116 and SeW_A1263, respectively), while serotypes Enteritidis, Gallinarum and Dublin encode different truncated forms of it (SEN1008/SEN1009, SG1050/SG1051 and SeD_A1237, respectively). Notably, SeAg_B1116 and SeW_A1263 share ~31–38% sequence identity with several secreted Sel1-like proteins of *L. pneumophila *involved in manipulation of vacuolar trafficking in macrophages [23].

**Figure 2:**
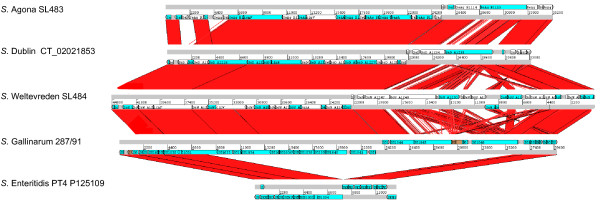
**Comparative analysis of SPI-19 in *Salmonella***. DNA-based comparison of SPI-19 in serotypes Agona strain SL483, Dublin strain CT_02021853, Weltevreden strain SL484, Gallinarum strain 287/91 and Enteritidis strain P125109. BLASTN analysis was performed using WebACT and displayed with the ACT software.

The genetic architecture of SPI-19 is highly conserved in serotypes Dublin, Weltevreden, Agona and Gallinarum. The main structural differences observed are circumscribed to the right end of the island, a region encoding a variable number of proteins with unknown function flanked by Rhs elements (Figure 2). Gene content variability in this region can be explained by deletions and rearrangements involving recombination events between Rhs elements. In serotype Gallinarum we identified a small ORF downstream of SG1040 that is absent in the genome annotation. This ORF encodes a gp25-like protein homolog to the T6SS-related ORF VCA0109 in *V. cholerae *(Additional file 2), and is annotated in the genome of the other SPI-19 positive serotypes.

In serotype Agona, the cluster of genes related to sugar metabolism flanking the left end of SPI-19 is missing. The absence of these genes has been reported previously [24]. The existence of 3 ORFs (SeAg_B1087 to SeAg_B1089) encoding transposase remnants at this junction may be related to the absence of these genes. In addition, SPI-19 in serotype Agona includes a third ImpA homolog (SeAg_B1094) adjacent to a truncated second copy of IcmF (SeAg_B1095). Probably, these ORFs were generated by a duplication of neighboring genes encoding complete versions of both ImpA and IcmF proteins (SeAg_B1098 and SeAg_B1099, respectively).

In serotype Enteritidis, SPI-19 presents an internal deletion of ~24 kb with respect to the island in serotypes Dublin, Weltevreden, Agona and Gallinarum (Figure 2). As the result of this deletion, the island in Enteritidis only encodes 16 ORFs, 3 of which correspond to T6SS core components: SEN1002 (a putative Hcp-1 protein), SEN1003 (an ImpA homolog) and SEN1004 (a truncated form of IcmF). The absence of essential components suggests that the T6SS encoded in this serotype is not functional. Interestingly, the internal region of SPI-19 absent in Enteritidis has been defined as the "region of difference 9" (ROD9) in a comparative genome analysis of serotypes Gallinarum and Enteritidis [25]. It is tempting to speculate that the presence of an active T6SS is somehow related to differences in host adaptation presented by these serotypes.

As mentioned before, SPI-19 is not obviously linked to a tRNA-encoding gene. Comparative genomic analyses revealed that SPI-19 is located at the left junction of a large chromosomal region (~900 to 960 kb, depending on the serotype) inverted with respect to the genome of serotype Typhimurium [25]. The right end of the inverted region is flanked by a functional copy of the tRNA-encoding gene *serX*. A more detailed sequence analysis revealed the presence of a 109 bp-long scar located at the left border of SPI-19 in the genomes of serotypes Dublin, Weltevreden, Gallinarum and Enteritidis. In serotype Agona, the last 48 nucleotides of the scar are lost. Notably, in each case the scar includes 15 nt in the 3' end of *serX*. Thus, a truncated version of *serX *is flanking the left end of SPI-19. These observations suggest that SPI-19 was originally inserted at the 3' end of *serX*, being flanked by a truncated and a functional copy of this tRNA-encoding gene. A posterior inversion event repositioned the functional copy of *serX *to a location distant from SPI-19 in the genome of serotypes Dublin, Weltevreden, Agona, Gallinarum and Enteritidis. It has been reported that insertions of new horizontally acquired genetic material could trigger compensating genomic rearrangements in order to maintain genome stability [26]. Thus, the acquisition of SPI-19 could be one of the events leading to the observed inversion. These events must have occurred after the divergence of serotype Typhimurium and a common ancestor of serotypes encoding SPI-19.

### Subspecies *arizonae *(IIIa) encodes two novel T6SS gene clusters

Besides SPI-6 T6SS and SPI-19, two additional T6SS loci were identified in *Salmonella*. The novel gene clusters, now designated SPI-20 and SPI-21, are present only in the genome of *S. enterica *subspecies *arizonae *(IIIa) serotype 62:z4,z23:-. Notably, SPI-6 and SPI-19 are not encoded in the genome of this serotype.

**Figure 3:**
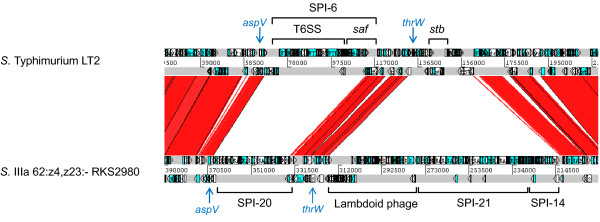
**Genomic context of SPI-20 and SPI-21 in *S*. IIIa 62:z4,z23:-**. DNA-based comparison of the genomic surrounding of SPI-20 and SPI-21 in *S*. IIIa 62:z4,z23:- strain RKS2980 and the corresponding region in the genome of *S*. Typhimurium strain LT2. BLASTN analysis was performed using WebACT and displayed with the ACT software. The location of key genetic elements is indicated. tRNA-encoding genes are indicated by blue arrows. *saf *and *stb *correspond to *Salmonella *fimbrial operons. SPI: *Salmonella *Pathogenicity Island.

SPI-20 corresponds to a ~34 kb genomic island encoding 28 ORFs (SARI_02707 to SARI_02736, coordinates 2617346-2651626) with an average G+C content of 53.1%. Seventeen ORFs related to T6SS function were identified, 13 of which correspond to core components (Figure 1 and Additional file 3). Among the T6SS-related components, the island encodes an Hcp-like protein (SARI_02729) and a VgrG protein with no C-terminal extension (SARI_02724). In addition, 3 ImpA homologs were identified: SARI_02718, SARI_02719 and SARI_02709 (Additional file 3). Sequence analyses revealed that SARI_02718 and SARI_02719 encode truncated proteins corresponding to the C-terminal and N-terminal of an ImpA homolog, respectively. Most probably, both ORFs were generated as the result of a point mutation that introduced a stop codon in SARI_02719. On the other hand, SARI_02709 encodes an ImpA homolog distantly related to SARI_02719/SARI_02718. SPI-20 encodes neither transcriptional/post-transcriptional regulators nor FHA-domain containing proteins present in other T6SS loci [11,20]. As in the case of SPI-19, the G+C content of SPI-20 is not uniformly distributed. While most of the island has a G+C content of 55–60%, regions with low G+C content (~42%) encoding proteins with unknown function were detected. Although SPI-20 is located in the proximity of the tRNA-encoding gene *aspV *(Figure 3), the island is not flanked by obvious direct repeats. Interestingly, *aspV *corresponds to the insertion site of SPI-6 in many serotypes of *S. enterica*, indicating that different T6SS have been acquired at the same genomic location during *Salmonella *evolution.

SPI-21 is a second island encoding a T6SS in the genome of serotype IIIa 62:z4,z23:-. This island has an extension of ~55 kb and is delimited by transposase remnants (Additional file 4). Again, the average G+C content of the island (49.6%) is unevenly distributed, presenting regions with low G+C content (41.3%) encoding hypothetical proteins of unknown function and regions with higher G+C content (54.3%) encoding T6SS-related ORFs. SPI-21 is flanked by 2 horizontally acquired genetic elements (Figure 3). Adjacent to its right junction, a cryptic lambdoid phage (SARI_02636 to SARI_02688) is inserted at the 3' end of *thrW*. This tRNA-encoding gene corresponds to the insertion site of P22 and related phages in *Salmonella *[27]. In addition, a small genomic island encoding 8 ORFs (SARI_2570 to SARI_02577) was found in the vicinity of the left junction of SPI-21. Five of these ORFs share extensive homology to ORFs in SPI-14 (STM0855 to STM0859 in serotype Typhimurium), initially described as a locus encoding genes required for full virulence of serotype Gallinarum in chickens [28].

**Figure 4:**
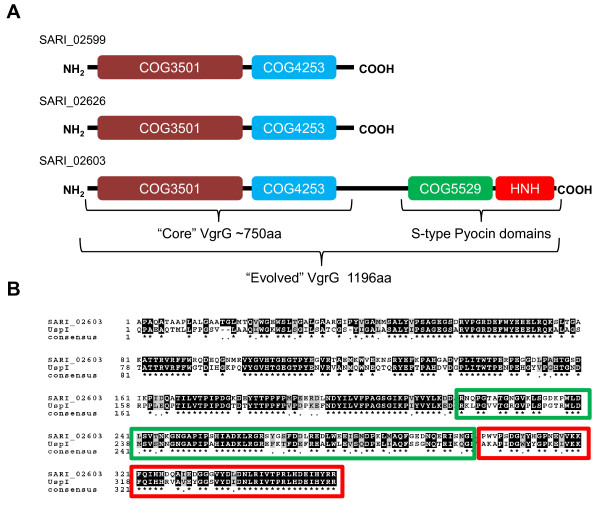
**VgrG homologs encoded in SPI-21**. **(A) **Schematic representation of three VgrG proteins encoded in SPI-21. Conserved protein domains are represented in color. **(B) **Homology between the C-terminal extension of SARI_02603 and Usp in UPEC strains. SARI_02603 [residues 841–1201 (1201 total)] and Usp [residues 241–593 (593 total)] were aligned using the multiple sequence alignment tool ClustalW2. Identical and conserved residues are indicated by shaded boxes. Consensus residues are indicated below alignment with an asterix. S-type pyocin and HNH domains are indicated in green and red color boxes, respectively.

SPI-21 includes 57 ORFs (SARI_02578 to SARI_02635, coordinates 2504768-2560524), 20 of which encode T6SS-related components (Figure 1 and Additional file 4). Among the ORFs not linked to T6SS, a putative chitinase (SARI_02620) and a protein (SARI_02608) containing the PASTA (penicillin-binding protein and Ser/Thr kinase associated) domain [29] were identified. Notably, SPI-21 presents additional ORFs linked to T6SS besides the core components shared with SPI-20. Hence, the island encodes not one but two VgrG proteins without C-terminal extension (SARI_02599 and SARI_02626). In addition, SPI-21 encodes an evolved VgrG protein (SARI_02603) containing a C-terminal extension that presents two conserved domains: COG5529 (S-type pyocin family) and PF01844 (HNH endonucleases) (Figure 4A). This constitutes one of the main findings of the present study as this is not only the first evolved VgrG described in *Salmonella*, but also the first evolved VgrG including a pyocin domain described so far in the literature. The C-terminal extension in SARI_02603 shares extensive sequence identity with the uropathogenic specific protein (Usp), a putative S-type pyocin encoded by UPEC strains [30] (Figure 4B). S-type pyocins are protease-sensitive, colicin-like bacteriocins produced by *P*. *aeruginosa *strains. These bacteriocins are normally constituted by two proteins: a large component that carries the killing activity and a small component that serves as an immunity protein [31]. Noteworthy, SPI-21 includes 4 ORFs encoding putative colicin/pyocin immunity proteins (SARI_02581, SARI_02585, SARI_02586 and SARI_02602), one of which is encoded just downstream of SARI_02603 (Additional file 4). Altogether, the presence of putative bacteriocin immunity proteins and an evolved VgrG with potential bacteriocin activity in SPI-21 suggests for the first time that bacteria could use a T6SS to kill other bacteria.

### The SPI-6 T6SS gene cluster is widely distributed in *Salmonella enterica*

As mentioned before, the only T6SS identified to date in *Salmonella *is located in SPI-6, a pathogenicity island that also encodes the Saf and Tcf fimbrial systems [19,32]. The presence of the T6SS associated to SPI-6 was confirmed in 16 of the genomes surveyed, all of them belonging to *S. enterica *subspecies *enterica *(I). This group includes serotypes Typhimurium, Typhi, Paratyphi A, Paratyphi C, Kentucky, Newport, Heidelberg, Saintpaul, Schwarzengrund, Tennessee, Hadar, Infantis, Choleraesuis, Weltevreden, Dublin and I 4, [5], 12:i:- (Table 1). The ORFs, product sizes, conserved protein domains and genome coordinates of SPI-6 T6SS genes in representative strains of the mentioned serotypes are detailed in Additional file 5. Furthermore, the presence of the *saf *fimbrial operon was also detected in each of these serotypes, and the additional presence of the *tcf *fimbrial operon was confirmed in serotypes Paratyphi A, Choleraesuis and Typhi. This indicates that the 16 serotypes encoding the T6SS within SPI-6 harbor a complete version of this pathogenicity island.

Depending on the serotype, SPI-6 T6SS corresponds to a region of ~35 to 50 kb that encodes ~30 to 45 ORFs, including each of the 13 T6SS core components (Figure 1 and Additional file 5). Noteworthy, critical T6SS components in many SPI-6 T6SS are encoded by pseudogenes, as the case of SciI (VipB homolog) in serotype Typhi, and SciG (ClpV homolog) in serotypes I 4,[5],12:i:- and Choleraesuis (Additional file 5). This observation suggests that these T6SS are not functional.

**Figure 5:**
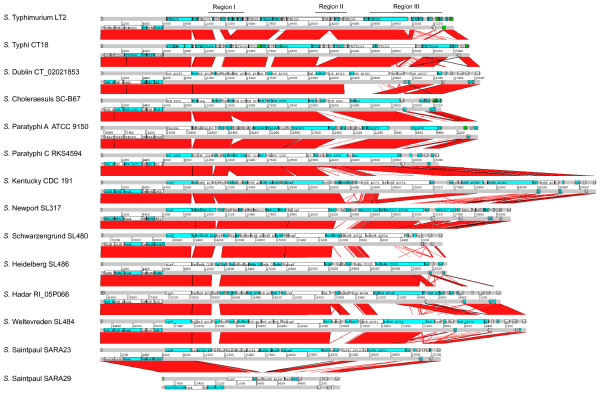
**Genetic architecture of SPI-6 T6SS in *Salmonella enterica *serotypes**. DNA-based comparison of SPI-6 T6SS in 13 different serotypes of *S. enterica *was performed by BLASTN analysis with WebACT and visualized with ACT software. The three major regions presenting structural differences between serotypes are indicated.

The genetic architecture of SPI-6 T6SS is highly conserved among serotypes (Figure 5 and Additional file 5). However, structural differences limited to 3 particular regions in the island were observed. The first region is located downstream of *sciI *(*vipB *homolog), and encodes a variable number of proteins with no known function and embedded Hcp-like proteins. The obvious consequence of such variability is the presence of either one or two copies of Hcp-like proteins in different serotypes. The second region is located downstream of *sciS *(*icmF *homolog) and encodes a variable number of proteins with unknown function. The third region is located downstream of *vrgS *(*vgrG *homolog) and encodes a variable number of Rhs elements. In contrast to SPI-19, the variability observed in this region is not explained by deletions involving recombination between Rhs elements. More likely, different serotypes have acquired a distinct repertoire of unrelated Rhs-like elements.

Differences in the genetic structure of SPI-6 T6SS among strains of the same serotype were only detected in the case of *S*. Saintpaul. In this serotype, strain SARA29 presents a ~15 kb deletion in SPI-6 with respect to strain SARA23 (Figure 5). As the result of this deletion, SPI-6 T6SS in SARA29 does not encode critical components such as SciS (IcmF homolog), SciI (VipB homolog) and the two Hcp-like proteins, suggesting that the only T6SS encoded in this strain is not functional.

The analysis also revealed that SPI-6 in serotypes Enteritidis, Gallinarum, Agona, Paratyphi B and Virchow does not encode a T6SS (Table 1), but maintain the *saf *fimbrial operon. Furthermore, SPI-6 in serotype Javiana neither encodes a T6SS nor a fimbrial operon. These observations support the notion that the T6SS and the associated fimbrial operons *saf *and *tcf *have been acquired independently during the evolution of *Salmonella *to produce a complex SPI-6. A more detailed sequence analysis revealed that SPI-6 in serotype Javiana only includes 5 ORFs, one of which corresponds to an ImpA-like protein associated to the SPI-6 T6SS. Several additional remnants of T6SS-related ORFs associated with SPI-6 were identified in serotypes Enteritidis, Gallinarum, Agona, Paratyphi B and Virchow (Additional file 6). The presence of these remnant ORFs suggests that an ancestor of SPI-6 T6SS was originally present in the genome of these serotypes, and was subsequently lost during evolution. In addition, a remnant of the first ORF in SPI-20 (and not linked to the T6SS function) was identified within SPI-6 in serotypes Gallinarum, Paratyphi B and Virchow (Additional file 6), suggesting that an ancestor of SPI-20 may have been present at this location. This hypothesis is supported by the fact that the insertion site for both SPI-6 and SPI-20 in *Salmonella *corresponds to the same tRNA-encoding gene, *aspV*.

### Organization of genes encoding core components in T6SS loci

Boyer and coworkers identified three groups of T6SS core components presenting a conserved genomic organization [8]. These conserved "modules" were defined by computing the number of loci in which every pair of T6SS core components (identified by their distinct COG ID) could be co-transcribed. The first module includes COG3521 (putative lipoprotein), COG3522, COG3455 (DotU) and COG3523 (IcmF). The second module includes COG3516 (VipA), COG3517 (VipB) and COG3157 (Hcp). The third module includes COG3518 (gp25-like protein), COG3519 (VasA) and COG3520 [8]. When we analyzed the conservation of these modules for each T6SS locus in *Salmonella*, important differences were identified:

• In the first module, components COG3521, COG3522 and COG3455 are linked in SPI-6 and SPI-19, while COG3523 is disconnected. In contrast, only COG3522 and COG3455 are linked in SPI-20 and SPI-21, while both COG3521 and COG3523 are disconnected. Furthermore, SPI-21 includes a second copy of COG3521 that is also unlinked to the proposed module.

• In the second module, COG3516 and COG3517 are linked in the 4 *Salmonella *T6SS loci, while COG3157 (Hcp) is not linked to either COG3516 or COG3517. In addition, both SPI-6 T6SS and SPI-19 encodes two Hcp proteins in different regions of each T6SS locus. Furthermore, in SPI-19 one of these ORFs (Hcp-1) is located adjacent to COG3516, but in a divergent orientation, clearly indicating that COG3516/COG3517 and Hcp have evolved independently. This is consistent with the report of Hcp proteins involved in T6SS function that are encoded outside the T6SS locus in *V. cholerae *[20], and also to the presence of "orphan Hcps" apparently unlinked to the T6SS function in *Salmonella *and other bacteria (see below).

• Finally, a conserved third module is present in SPI-6 T6SS and SPI-19. On the other hand, while COG3519 and COG3520 are linked in SPI-20 and SPI-21, COG3518 is disconnected from the module. Furthermore, SPI-21 includes 2 additional copies of COG3518 that are also disconnected from the module, and one from each other.

The similarities detected between modules in SPI-20 and SPI-21 indicate a close phylogenetic relationship, while differences between modules in SPI-6 T6SS, SPI-19 and SPI20/SPI-21 suggest that these loci have been acquired through independent horizontal transfer events, or have evolved independently to perform specialized functions.

### Comparative analysis of *Salmonella *T6SS gene clusters

A basic comparative analysis of the *Salmonella *T6SS loci described in this study revealed that they share very limited homology one to each other at the DNA level (Additional file 7). This result supports the notion that these genomic islands were acquired by independent horizontal gene transfer events during *Salmonella *evolution. To identify evolutionary relationships between these T6SS gene clusters, we followed the approach used by Bingle and coworkers to study phylogeny between T6SS loci [1]. The concatenated aminoacidic sequences of conserved components VipA and VipB from 66 T6SS loci (including the original 37 loci used by Bingle and coworkers [1] and 22 loci representing every T6SS in *Salmonella*) were analyzed.

**Figure 6:**
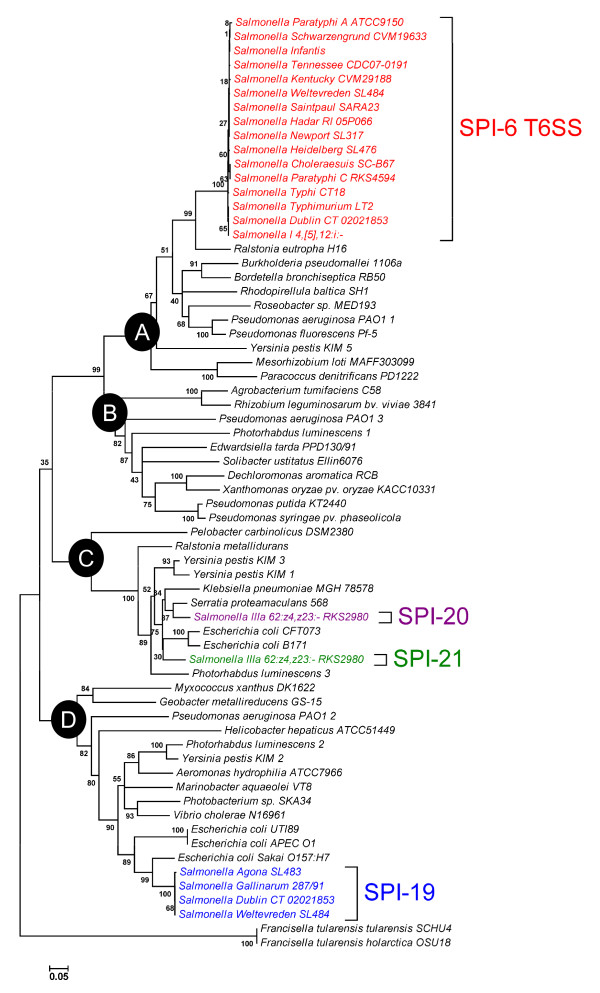
**Evolutionary relationships of *Salmonella *T6SS loci**. A distance tree (neighbour-joining) was calculated from concatenated VipA and VipB protein sequences of previously identified T6SS gene clusters, including the 3 novel *Salmonella *T6SS loci. Each of the four major phylogenetic groups is shown in the nodes labeled A to D. Bootstrap support values (% from 3,000 replicates) were: A, 99%; B, 80%; C, 99% and D, 99%.

Notably, the T6SS loci in *Salmonella *differentially clustered in the mayor phylogenetic groups described for T6SS gene clusters [1] (Figure 6). Thus, every SPI-6 T6SS locus included in our analysis belongs to Group A, also including the T6SS gene clusters *h16_A0645*-*h16_A0657 *in *Ralstonia eutropha *strain H16 and *tss*-2 in *Burkholderia pseudomallei *strain 1106a. This result agrees with previous reports on the evolutionary relationship between SPI-6 T6SS in Typhimurium and *tss*-2 [1,8]. In addition, SPI-20 and SPI-21 belong to Group C, which includes the T6SS cluster within PAI-*metV *in the uropathogenic *E. coli *(UPEC) strain CFT073. Finally, each SPI-19 analyzed belongs to Group D, also including the T6SS cluster within OI#7 in *E. coli *O157:H7 strain Sakai, and the AGI-1 island in the avian pathogenic *E*. *coli *(APEC) strain O1 (Figure 6). According to the nomenclature established in a recent phylogenetic analysis performed by Boyer and coworkers [8], SPI-6 T6SS, SPI-19 and SPI-20/SPI-21 belong to sub-groups III, I and II, respectively.

**Figure 7:**
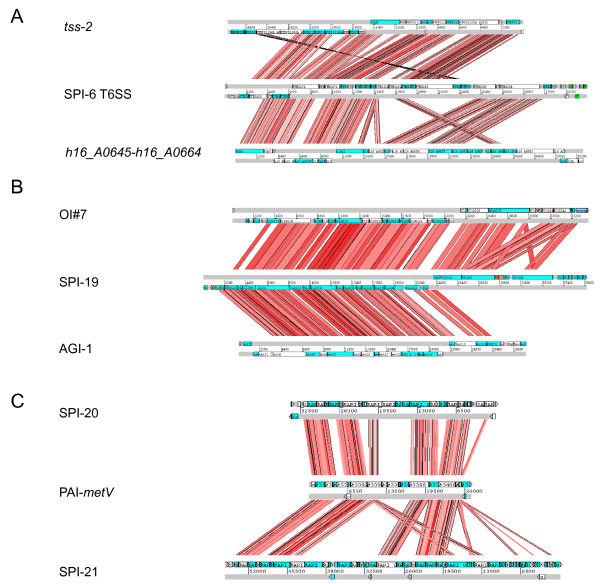
**Comparative analysis of *Salmonella *T6SS clusters**. DNA-based comparison of the T6SS encoded in SPI-6, SPI-19, SPI-20 and SPI-21 and phylogenetically-related T6SS loci. The analysis was performed by BLASTN with WebACT and visualized with ACT software. **(A) **Comparison of SPI-6 T6SS in serotype Typhimurium strain LT2 with gene cluster *tss-2 *in *Burkholderia pseudomallei *strain 1106a and *h16_A0645*-*h16_A0657 *in *Ralstonia eutropha *strain H16. **(B) **Comparison of SPI-19 in serotype Gallinarum strain 287/91 with the OI#7 island in *Escherichia coli *O157:H7 strain Sakai and island AGI-1 in APEC strain O1. **(C) **Comparison of SPI-20 and SPI-21 in *S*. IIIa 62:z4,z23:- strain RKS2980 with the T6SS gene cluster within PAI-*metV *in UPEC strain CFT073.

In a second analysis, we determined identity at the protein level of each *Salmonella *T6SS gene cluster, as a whole, to the closest phylogenetic relatives identified by our previous analysis. Notably, SPI-6 T6SS presented extensive identity and shared a similar genetic architecture with T6SS gene clusters *tss*-2 in *B. pseudomallei *and *h16_A0645*-*h16_A0657 *in *R*. *eutropha *(Figure 7A). The same was true for SPI-19 and the whole T6SS gene cluster within OI#7 in EHEC and the AGI-1 island in APEC (Figure 7B). On the other hand, SPI-20 and SPI-21 present several differences with its relative T6SS within PAI-*metV *in UPEC (Figure 7C). Thus, although SPI-20 conserves the overall genetic architecture of the T6SS locus in UPEC, the island includes several additional ORFs encoding proteins of unknown function (Additional file 3). In addition, SPI-21 presents a genetic architecture similar to SPI-20 and to the T6SS gene cluster in PAI-*metV*. Nevertheless, the island includes additional embedded ORFs encoding multiple copies of VgrG and gp25-like proteins, putative transposases, a putative chitinase and colicin/pyocin immunity proteins (Additional file 4). In spite of this, the phylogenetic proximity of SPI-20 and SPI-21 is evident.

The results obtained from both phylogenetic analyses reached the same conclusions, validating the evaluation of divergence in the aminoacidic sequence of VipA and VipB homologs to determine phylogenetic relationships between different T6SS loci. Altogether, these results strongly suggest that SPI-6 T6SS, SPI-19 and SPI-20/SPI-21 were acquired by independent horizontal transfer events from several unrelated bacterial species during the evolution of *Salmonella*. This is supported by the following observations: (i) each cluster is encoded in or corresponds to a genomic island that includes every T6SS core component, (ii) SPI-6 T6SS and SPI-20 are located adjacent to the tRNA-encoding gene *aspV*, a known hot-spot for insertion of genetic elements by horizontal transfer, (iii) SPI-19 is linked to a large chromosomal inversion, presumably caused by its insertion in the tRNA-encoding gene *serX*, and (iv) SPI-6 T6SS, SPI-19 and SPI-20/SPI-21 belong to different T6SS phylogenetic groups (Figure 6). Finally, the similar genetic organization presented by SPI-20 and SPI-21 (Figure 1), the presence of conserved regions sharing extensive sequence identity at the protein level (Figure 7), and the fact that they belong to the same T6SS phylogenetic group (Figure 6) support a common origin for these T6SS loci. Thus, the presence of SPI-20 and SPI-21 in the genome of *S*. IIIa serotype 62:z4,z23:- can be explained either as the result of the acquisition of one of these clusters by lateral transfer and a posterior duplication event, or by sequential lateral transfer of each island from closely related donor organisms.

We can hypothesize evolutionary origins for each T6SS locus in *Salmonella *considering both phylogenetic analyses performed. Thus, SPI-6 T6SS may have been acquired from a beta-proteobacteria in the *Burkholderiaceae *family, related to *Burkholderia pseudomallei *and *Ralstonia eutropha *(recently reclassified as *Cupriavidus necator*). In contrast, SPI-19 seems to have been acquired from a pathogenic *E. coli *strain related to EHEC and APEC. Finally, SPI-20 and SPI-21 may have been acquired from a UPEC strain. In any case, each T6SS locus seems to have further evolved independently to generate systems performing specialized functions.

### Orphan Hcp-like proteins in *Salmonella*

Several ORFs encoding additional Hcp-like proteins in *Salmonella *were identified in this study (Additional file 8). Because these proteins are not directly linked to T6SS gene clusters, we referred to them as "orphan Hcp-like proteins". According to their distribution, these proteins can be attributed to three major groups: (i) those present in every *S. enterica *serotype analyzed (e.g., STM4509.s and STM3131 in serotype Typhimurium), (ii) an unique representative present only in serotypes Agona, Paratyphi A, Kentucky and Typhi (e.g, SeAg_B3284 in serotype Agona), and (iii) those present only in *S*. IIIa serotype 62:z4,z23:- (i.e., SARI_00061, SARI_00912, SARI_01363 and SARI_03217). The genomic location and distinctive features of each of these proteins are detailed in Additional file 8.

Homologs of STM4509.s and STM3131 are widely distributed among *S. enterica *serotypes. Noteworthy, STM4509.s corresponds to HilE, a 148 aa protein that negatively regulates the expression of *hilA*, which encodes the master regulator controlling the expression of SPI-1 T3SS in *Salmonella *[33]. Folkesson and coworkers were the first to identify a similarity between HilE and Hcp proteins encoded in SPI-6 [19]. On the other hand, STM3131 corresponds to a 161 aa protein with no known function encoded in SPI-13, a locus including genes required for full virulence of serotypes Enteritidis, Gallinarum and Typhimurium in different animal hosts [28,34,35]. The genes encoding STM3131 and STM3130 constitute a small operon predicted to be part of the PmrAB regulon [36]. Remarkably, STM3130 belongs to a family of proteins including the baseplate gp25 protein in phage T4 and relatives of a conserved T6SS core component [8].

As mentioned, an orphan Hcp-like protein present only in serotypes Kentucky, Paratyphi A, Agona and Typhi was also identified. Depending on the serotype, this orphan Hcp corresponds to a protein of either 40 or 66 aa. In all cases, this small ORF is encoded in SPI-8 and is located downstream of an integrase remnant in the vicinity of the tRNA-encoding gene *pheV*. Finally, *S*. IIIa 62:z4,z23:- harbors four additional orphan Hcp-like proteins, each of them placed adjacent to transposase remnants or regions acquired by lateral gene transfer. Notably, SARI_03217 corresponds to an unusually long Hcp-like protein (403 aa versus ~160 aa in regular Hcp proteins) containing the Hcp/COG3157 domain at the N-terminus (150 aa) and a C-terminal extension (253 aa) including no conserved protein domain.

There are reports of other unusually long Hcp proteins carrying C-terminal extensions due to acquisition of novel protein domains. This is the case of Usp, a putative bacteriocin related to S-type pyocins containing an Hcp domain at the N-terminus [30]. To date, there is no experimental evidence confirming the putative bactericidal activity of Usp or explaining the role played by the Hcp domain in its function. However, the *usp *gene has been widely used as a virulence marker in UPEC strains [37-39]. Another example corresponds to the hypothetical protein YhhZ of *E. coli*. This protein also contains an Hcp domain at the N-terminus and a degenerate HNH domain at the C-terminus. The *yhhZ *gene has been reported to be induced during growth of *E. coli *in biofilms [40]. The structural features presented by these long Hcp proteins (i.e. an Hcp/COG3157 domain linked to an additional C-terminal domain) resemble those presented by the evolved VgrG proteins. Because of this similitude, we propose to designate these proteins as "evolved Hcps". Apparently, both Usp and YhhZ correspond to evolved Hcps not linked to T6SS function and seem to serve different roles for the bacterial cell. This could also be the case for SARI_03217 in *S*. IIIa 62:z4,z23:-.

**Figure 8:**
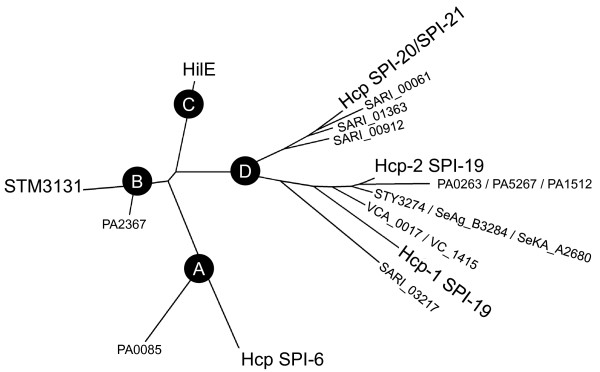
**Evolutionary relationships of Hcp-like protein**. A distance tree (neighbour-joining) was calculated from the alignment of every Hcp-like protein identified in *Salmonella*. The analysis also included Hcp proteins of *Pseudomonas aeruginosa *strain PA01 and *Vibrio cholerae *strain V52. Each of the major groups of Hcp phylogeny is shown in the nodes labeled A to D. Bootstrap support values (% from 3,000 replicates) were: A, 98%; B, 94%; C, 98% and D, 80%.

In order to identify evolutionary relationships between these orphan Hcp-like proteins and those directly linked to T6SS loci, the sequence divergence among these proteins was analyzed. As a reference, 5 Hcp proteins described in *P. aeruginosa *strain PA01 (PA0263, PA1512, PA5267, PA2367 and PA0085) and 2 described in *V. cholerae *strain V52 (VCA_0017 and VC_1415) were included in the analysis. The results revealed the presence of 4 groups of Hcp relatives (Figure 8). Group A includes PA0085 and every Hcp protein encoded in SPI-6 T6SS. Group B includes PA2367 and every STM3131 homolog. Group C includes only HilE homologs, and Group D includes PA1512, PA5267, PA0263, VCA_0017, VC_1415, Hcp proteins in SPI-19, SPI-20, SPI-21, and the orphan Hcp-like proteins in *S*. IIIa 62:z4,z23:- (Figure 8).

Although evolutionary relationships between Hcp-like proteins were determined, the analysis failed to predict a role for many of them in T6SS function. We believe that this reflects the fact that the Hcp domain is linked to a particular folding pattern or structure rather than to a specific function or activity. Altogether, our data support the notion that SARI_03217 and HilE homologs arose from a common ancestor of every Hcp protein and further evolved to gain new functions, while keeping structural properties of its relatives directly linked to T6SS function.

## Conclusion

This study expands the current knowledge on bacterial type VI secretion by the identification and description of 3 novel T6SS loci in *Salmonella*. We have determined that each T6SS locus has a distinct evolutionary history, indicating acquisition through independent horizontal gene transfer events. Interestingly, while some serotypes harbor multiple T6SS loci others do not encode for any. This observation indicates that T6SS function is not essential for virulence but may provide an additional advantage in certain environments. On the other hand, the presence of 2 T6SS in many serotypes perhaps reflects specific roles for each of them in different steps during infection or in different environments or hosts. Our analysis also identified an evolved VgrG protein that contains a C-terminal extension sharing identity to S-type pyocins. To the best of our knowledge, this is the first description of a VgrG protein carrying a bacteriocin effector domain and suggests a novel role for T6SS in bacterial killing. The functionality of the 4 T6SS described here and their roles in *Salmonella *pathogenesis and host-specificity are currently under study in our laboratory.

## Methods

### *Salmonella *genomic data acquisition

Information about the current status of *Salmonella *genome sequencing projects was obtained from the Genomes On Line Database (GOLD; updated on May 2, 2009) ([41], web site: http://www.genomesonline.org/) and from Table 1 in reference [42]. The 44 *Salmonella *sequencing projects having sequence data publicly available were selected for genome-wide *in silico *identification of T6SS loci. The genomes analyzed covered 24 different serotypes of *Salmonella enterica *(22 in subspecies I, 1 in subspecies IIIa and 1 in subspecies IIIb). Genome accession numbers and information about the *Salmonella *genome sequencing projects utilized in this work are detailed in the Additional file 1.

### *In silico *identification of T6SS loci

Nucleotide sequence of ORFs representing T6SS core components were obtained from public sequence databases. Three representatives of each branch in T6SS phylogenetic trees previously described [1,8] (Additional file 9) were used as baits in sequential BLASTN, BLASTP and TBLASTX searches [43-45] to identify T6SS homologs in the 44 *Salmonella *genomic sequences listed in Additional file 1. When a core component was identified, a systematic analysis was performed to detect adjacent T6SS components. A T6SS locus was defined as a gene cluster encoding at least 5 core components. Boundaries of the genomic islands encoding T6SS were defined by thorough nucleotide sequence analysis. A systematic analysis of gene content and genetic architecture of the whole T6SS gene clusters identified was performed for 31 annotated and fully assembled genomes. The remaining 13 genomes correspond to unfinished projects; therefore in many cases an exhaustive ORF-by-ORF analysis of T6SS genetic architecture was not possible. In this case, we only determined the presence/absence status for T6SS core components in unassembled contigs.

### DNA and protein sequence analysis

Nucleotide sequences were analyzed by the sequence visualization and annotation tool Artemis version 10 [46] and the Vector NTI Advanced software version 11.0 (Invitrogen). Graphical representations of T6SS gene clusters were generated with the CLC Sequence Viewer software version 6.0.2 (CLC bio). For comparative analysis, nucleotide sequences were aligned by BLASTN and TBLASTX with the WebACT online resource ([47], web site: http://www.webact.org/) and visualized with the Artemis Comparison Tool (ACT) release 6 [48]. Presence of conserved protein domains in each ORF encoded in the identified T6SS loci was determined by cross-reference of several publicly available databases: The Simple Modular Architecture Research Tool (SMART) ([49], web site: http://smart.embl.de/), the Conserved Domain Database and Search Service version 2.16 ([50], web site: http://www.ncbi.nlm.nih.gov/Structure/cdd/cdd.shtml) and the Clusters of Orthologous Groups of proteins (COGs) database ([51], web site: http://www.ncbi.nlm.nih.gov/COG/).

### Phylogenetic analyses

Phylogenetic analyses were performed with the Molecular Evolutionary Genetics Analysis (MEGA) software version 4.0.2 [52] based on the approach described by Bingle and coworkers [1]. In the case of T6SS loci, the concatenated aminoacidic sequences of VipA and VipB homologs encoded in 66 T6SS loci (Additional file 10) were aligned using ClustalW [53] with the default parameters. A similar alignment was carried out using 113 Hcp-like proteins from strains representing each serotype of *Salmonella *studied, *Pseudomonas aeruginosa *strain PA01 and *Vibrio cholerae *strain V52 (Additional file 8). Phylogenetic trees were built from the alignments by the bootstrap test of phylogeny (3,000 replications) using the neighbor-joining method with a Poisson correction model.

## Authors' contributions

CB, CS and IC conceived the study. CB acquired the genomic sequences and performed most of the bioinformatic and phylogenetic analyses. CS carried out bioinformatic and phylogenetic analyses. JJ carried out bioinformatic analyses and helped in acquisition of genomic sequences. CB and CS interpreted the data generated. CB, CS and IC drafted the manuscript. CB and JJ prepared figures, tables and additional files presenting the data. All authors read and approved the final manuscript.

## Supplementary Material

Additional file 1 ***Salmonella *genome sequencing projects utilized in this work**. This file includes information on the 44 *Salmonella *genome sequencing projects used in this report. Click here for file

Additional file 2 **Gene content of SPI-19 in different *Salmonella *serotypes**. This file includes the genomic coordinates, product sizes and conserved protein domains of every ORF encoded in SPI-19 in Enteritidis strain P125109, Gallinarum strain 287/91, Agona strain SL483, Dublin strain CT_02021853 and Weltevreden strain SL484. In addition, each component is compared to the corresponding homolog in the OI#7 T6SS gene cluster of *Escherichia coli *O157:H7 strain Sakai. Click here for file

Additional file 3 **Gene content of SPI-20 in *Salmonella *IIIa 62:z4,z23:-**. This file includes the genomic coordinates, product sizes and conserved protein domains of every ORF encoded in SPI-20 in *Salmonella *IIIa 62:z4,z23:- strain RKS2980. In addition, each component is compared to the corresponding homolog in the T6SS gene cluster within PAI-*metV *in UPEC strain CFT073. Click here for file

Additional file 4 **Gene content of SPI-21 in *Salmonella *IIIa 62:z4,z23:-**. This file includes the genomic coordinates, product sizes and conserved protein domains of every ORF encoded in SPI-21 in *Salmonella *IIIa 62:z4,z23:- strain RKS2980. In addition, each component is compared to the corresponding homolog in the T6SS gene cluster within PAI-*metV *in UPEC strain CFT073. Click here for file

Additional file 5 **Gene content of SPI-6 T6SS in different *Salmonella *serotypes**. This file includes the genomic coordinates, product sizes and conserved protein domains of every ORF encoded in SPI-6 T6SS in different *Salmonella enterica *serotypes. In addition, each component is compared to the corresponding homolog in serotype Typhimurium strain LT2. Click here for file

Additional file 6 **Presence of T6SS-related ORFs associated with SPI-6 T6SS and SPI-20 in *Salmonella *serotypes encoding remnants of these islands**. This document presents information about ORFs related to SPI-6 T6SS and SPI-20 in serotypes that do not encode these T6SS gene clusters. Reference ORFs for SPI-6 T6SS and SPI-20 were obtained from *S*. Typhimurium strain LT2 and *S*. IIIa 62:z4,z23:- strain RKS2980, respectively. Click here for file

Additional file 7 **Comparative analysis of T6SS gene clusters encoded in *Salmonella***. This figure shows a DNA-based comparison of the 4 T6SS loci of *Salmonella*. BLASTN analysis of one representative of each genomic island (SPI-6 T6SS, SPI-19, SPI-20 and SPI-21) was performed using WebACT and visualized with ACT software. Click here for file

Additional file 8 **Hcp-like proteins in *Salmonella***. This file includes a list of every Hcp-like protein in *Salmonella *and reference Hcp proteins in *Pseudomonas aeruginosa *and *Vibrio cholerae *used in phylogenetic analysis. Click here for file

Additional file 9 **T6SS components used as baits in genome-wide screens for novel T6SS loci in *Salmonella***. This table includes a list of T6SS core components in different phylogenetic groups that were used in this study as baits to identify novel *Salmonella *T6SS loci by *in silico *analyses. Click here for file

Additional file 10 **VipA/VipB homologs utilized to perform T6SS phylogenetic analysis**. This file includes a list of VipA/VipB homologs in 66 T6SS loci used in this study to identify T6SS phylogeny. Click here for file
