# Supplementary material for: Comparative genomic analysis uncovers 3 novel loci encoding type six secretion systems differentially distributed in Salmonella serotypes
Source: BMC Genomics. 2009 Aug 4;10:354. doi: 10.1186/1471-2164-10-354 (PMC2907695; doi:10.1186/1471-2164-10-354)
Supplement: Additional file 7 — Comparative analysis of T6SS gene clusters encoded in Salmonella. This figure shows a DNA-based comparison of the 4 T6SS loci of Salmonella. BLASTN analysis of one representative of each genomic island (SPI-6 T6SS, SPI-19, SPI-20 and SPI-21) was performed using WebACT and visualized with ACT software. [file 1471-2164-10-354-S7.pdf]

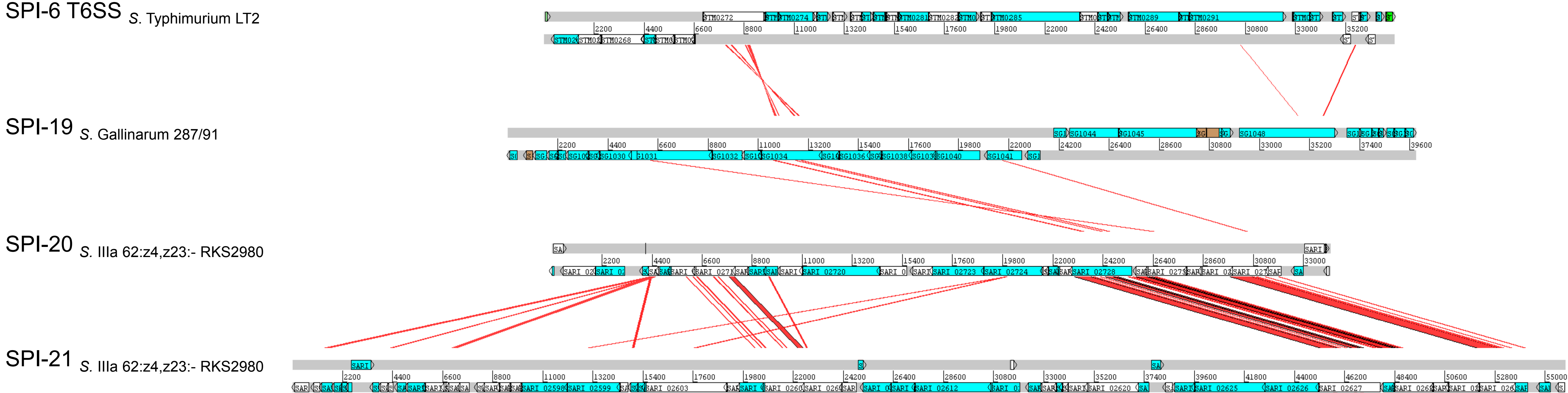
SPI-6 T6SS *S.* Typhimurium LT2
SPI-19 *S.* Gallinarum 287/91
SPI-20 *S.* IIIa 62:z4,z23:- RKS2980
SPI-21 *S.* IIIa 62:z4,z23:- RKS2980
